# Functionalized Buckyballs for Visualizing Microbial Species in Different States and Environments

**DOI:** 10.1038/srep13685

**Published:** 2015-09-08

**Authors:** Qingsu Cheng, Ashwin Aravind, Matthew Buckley, Andrew Gifford, Bahram Parvin

**Affiliations:** 1 Department of Electrical and Biomedical Engineering, University of Nevada, Reno, 1664 N Virginia Street, Reno NV, 89503, USA; 2 Life Sciences Division, Lawrence Berkeley National Laboratory, 1 Cyclotron Road, MS-977, Berkeley CA, 94720, USA; 3 Brookhaven National Laboratory, Bioscience Department, Bldg490, Upton, NY 11973, USA

## Abstract

To date, *in situ* visualization of microbial density has remained an open problem. Here, functionalized buckyballs (*e.g.*, C60-pyrrolidine tris acid) are shown to be a versatile platform that allows internalization within a microorganism without either adhering to the cell wall and cell membrane or binding to a matrix substrate such as soil. These molecular probes are validated via multi-scale imaging, to show association with microorganisms via fluorescence microscopy, positive cellular uptake via electron microscopy, and non-specific binding to the substrates through a combination of fluorescence and autoradiography imaging. We also demonstrate that cysteine-functionalized C60-pyrrolidine tris acid can differentiate live and dead microorganisms.

Soil hosts most of the biodiversity in the environment, where each cubic centimeter of soil matrix can contain hundreds of thousands of microorganisms that cohabitate in a complex assemblage of mineral and organic matter^1^. The structure and function of microbial communities are dynamic and play important and beneficial roles in productivity of ecosystems^2^, including oxygen production^3^, crop growth^4^, bioremediation^5^, carbon sequestration^6^, nitrogen fixation^7^, and water purification^8^. Simultaneously, microbial species may act as pathogens for living organisms. For example, plants from hundreds of different species are killed annually in Australia by *P. cinnamomi*^9^,^10^; and grain development, in wheat, is affected by infection of *G. graminis* var. *tritici* in vascular tissue^11^. Therefore, it is important to develop probes and assays that enable studying microbial species in their native environment, *i.e.*, *in situ* imaging. Applications of *in situ* imaging include, but are not limited to, the insights and understanding of the (i) composition and population of a normal gut microbiome as a function of exposure to antibiotics^12^ and/or under environmental stress; (ii) interactions and cross talk between microbes and plant roots in rhizosphere^13^,^14^; (iii) localization of endophytes in healthy plant tissues for improved yield^15^; and (iv) profiling of the microbial communities in soil crust for erosion control, water retention, and nutrient cycling^16^,^17^.

To meet the requirements of *in situ* imaging, synthesized probes must (i) penetrate the cell wall and lipid membrane, (ii) be non-sticky to the soil matrix, and (iii) differentiate between living and dead microorganisms. In a previous study^18^, our lab demonstrated that guanidinium-rich molecular transporters (GR-MoTrs) can internalize in different strains of algae by crossing both the cell wall and the lipid membrane; we later learned, however, that these molecular transporters are sticky to the matrix substrate. Other polymer-based nanoparticles, such as lipofectamine, have also been found to be sticky to the natural environment^19^. Moreover, in some cases, synthesized probes ideally should facilitate radiolabeling to meet the general requirements of *in situ* imaging. Although the current resolution of the imaging instruments for radiolabeled microbes is less than adequate, such limitations should not hinder probe synthesis since (a) specialized experiments can be designed for limited validation, and (b) imaging instruments will continue to evolve and improve. For example, the structure of a microbial community can be imaged with x-ray microtomography and MRI, but these techniques are destructive and do not report biological activities, the successful imaging of which is highly dependent on the design of the imaging instruments^20^,^21^.

Our approach for visualizing microorganisms with the potential ability to monitor microbial activities in soil matrix *in situ* is based on a fullerene C60 derivative: C60-pyrrolidine tris acid, a spherical multi-carbon molecule that is known better as a buckyball. Due to its versatile properties, fullerene has inspired the biology and chemistry scientific communities. It has been utilized as an antioxidant^22^, neuro-protective agent^23^, enzyme inhibitor^24^, anti-HIV reagent^25^, osteoporosis cure^26^, and radiotracer^27^. In addition, there are numerous applications of its variants, such as carbon nanotubes (CNTs)^28^, megatubes^29^, nano onions^30^, and fullerene rings^31^,^32^. These probes can be functionalized with a fluorescent tag for imaging microbial species, using fluorescence microcopy as one of the needed steps for validation and characterization (*e.g.*, localization, uptake). Our hypothesis is that C60-derivatives are capable of overcoming the major hurdles that were stated earlier, and removing barriers associated with monitoring the dynamics of microbial communities. In short, C60 derivatives can be functionalized per specific requirements. For example, functionalized C60 can be tagged and transported across the microbial cell wall and the lipid membrane to allow for localization and uptake studies; functionalized C60s are also non-sticky to the soil matrix, and can be washed out to reduce the background signal significantly. Our hypothesis has been validated using fluorescent and radioactive labeling of C60-pyrrolidine tris acid with multiple soil matrices and microbial species. The net result is that (a) experiments can be designed to assess viability and density of microorganisms dynamically, and (b) initiating the design of new imaging instruments can be better justified for visualizing microbial species in an opaque soil environment.

## Results and Discussion

**Figure 1:**
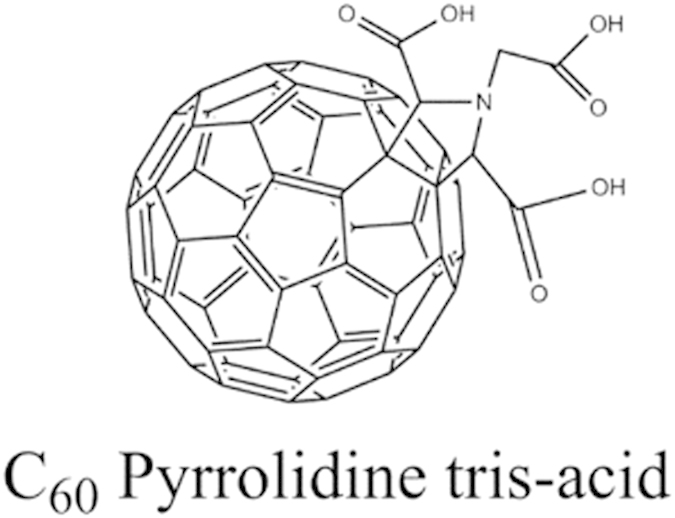
Schematic of C60-pyrrolidine tris acid, which indicates that C60-pyrrolidine tris acid has three potential decoration sites while maintaining the carbon cage structure intact.

C60-pyrrolidine tris acid is a derivative of fullerene C60 (Fig. 1) and possesses three key properties: (i) containing three carboxyl groups that allow for further decoration (*e.g.*, fluorescent tagging and radioactive isotope labeling); (ii) being extremely small (1–2 nm for a single molecule, 10–20 nm for a cluster of molecules), which facilitates intercellular movement and actions; and (iii) maintaining an intact carbon cage that retains enough hydrophobicity to inhibit adherence of C60-pyrrolidine tris acid to the soil matrix and organic matter. These properties have persuaded us to evaluate C60 as both a fluorescent and radiotracer reporter, where the rationale for functionalization is summarized below.

(i) Functionalization of C60-pyrrolidine tris acid with fBSA is based on EDC/NHS coupling that activates the carboxylic group, where fBSA replaces the NHS ester to form a stable conjugate. It has already been established that amine coupling through EDC/NHS is effective and practical in an aqueous phase^33^, and it is widely used in bio-related functionalization^34^,^35^,^36^. In our previous research^37^, we demonstrated that this method forms a CNT-PLGA-Caspase 3 conjugate, which in turn forms the basis for preparing C60-pyrrolidine tris acid-fBSA.

(ii) Functionalization of C60-pyrrolidine tris acid with ^14^C is based on the methylation of the carboxyl group with potassium hydroxide/dimethyl sulfate and radioactive methyl iodide. This method has been widely used for methylation of carboxylic acids and phenols^38^, where methylated C60 and C70 was demonstrated more than 20 years ago^39^,^40^. The final yield of ^14^C-methylated C60-pyrrolidine tris acid is about 20%.

(iii) Functionalization of C60-pyrrolidine tris acid with ^125^I uses the Finkelstein reaction, which has been widely described in the existing literature^41^,^42^,^43^. First, C60-pyrrolidine tris acid is functionalized with Cl by using N-chlorosuccinimide. Second, Cl is replaced by ^125^I through the Finkelstein reaction. The final yield of our ^125^I-labeled C60-pyrrolidine tris acid is about 20%.

### Microbial Internalization of C60-pyrrolidine tris acid

Internalization of fullerene materials has already been studied and demonstrated in mammalian systems. For example, CNTs have been shown to transport caspase-3 into osteosarcoma cells^37^, and C60 decorated with poly(ethylenimine) (PEI) has been used for cancer therapy^44^. However, little is known about whether C60 could transport cargo across the microbial cell wall. Therefore, to visualize whether C60-pyrrolidine tris acid can internalize within microorganisms, we have introduced a three-step validation protocol that involves fluorescence microscopy, transmission electron microscopy (TEM), and autoradiography. The model organisms include both Gram-negative and Gram-positive bacteria.

**Figure 2:**
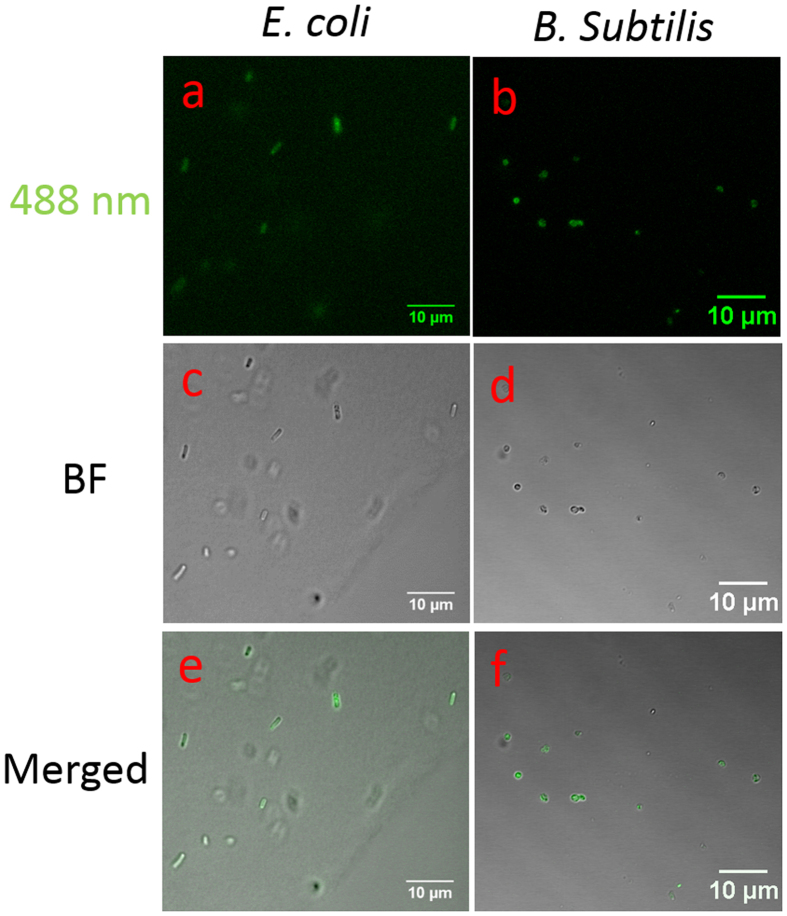
Cellular uptake of fBSA labelled C60-pyrrolidine tris acid by *E. coli* and *B. subtilis* monitored by Confocal Laser Scanning Microscopy. (**a**) and (**b**) fBSA-C60 pyrrolidine tris acid uptake by *E. coli* (**a**) and *B. subtilis* (**b**) visualized by exciting fBSA fluorescence using the 488 nm laser. (**c**) and (**d**) Bright field (BF) images of the microorganisms. (**e**) and (**f**) Merged fluorescence and BF images indicate that the fluorescent signals co-localize with the presence of the microorganisms.

*E. coli* (Gram-negative) and *B. subtilis* (Gram-positive) are incubated with functionalized C60-pyrrolidine tris acid with fBSA for 30 min. Samples are then washed with DI H_2_O to remove excess probes, and samples are then imaged by confocal microscopy. Figure 2 indicates a positive association of C60-pyrrolidine tris acid with microorganisms, where in Fig. 2a,b, both *E. coli* and *B. subtilis* have fluorescent signal emission following excitation by a 488 nm laser. We confirmed that these fluorescent signals are solely from C60-pyrrolidine tris acid-fBSA, because neither *E. coli* nor *B. subtilis* has an auto-fluorescence signal under the same conditions in the absence of fluorescent C60 (Supplementary Figure 1). In addition, these fluorescent signals co-localize with *E. coli* and *B. subtilis* cells by combining bright field and fluorescent imaging, which suggests either internalization within the cell or binding to the cell wall. (Recall that CNTs were able to internalize inside the mammalian cells^37^, and similar behavior can be suggested for microbial species).

**Figure 3:**
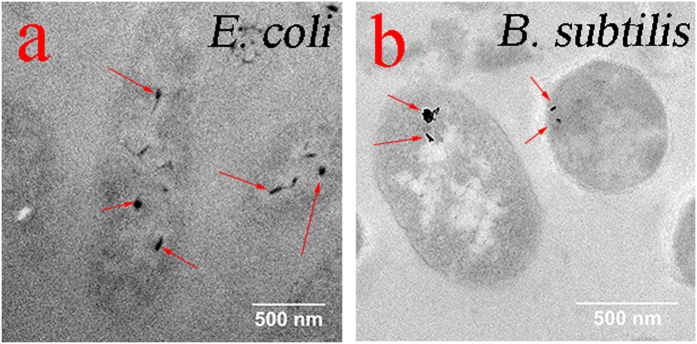
Cellular uptake of C60-pyrrolidine tris acid by *E. coli* and *B. subtilis* monitored by Transmission Electron Microscopy. The dark spots, pointed by the red arrows, represent C60-pyrrolidine-tris acid uptake by *E. coli* (**a**) and *B. subtilis* (**b**).

To test the hypothesis that functionalized C60-pyrrolidine tris acid internalizes within the cell, microorganisms are imaged with TEM. Both *E. coli* and *B. subtilis* are incubated with C60-pyrrolidine tris acid, washed to remove excess compound, sectioned into slices of 60 nm thickness, and then imaged by TEM. Figure 3 shows that C60-pyrrolidine tris acid localizes within the cell in both *E. coli* and *B. subtilis*. The control experiment consists of (i) C60-pyrrolidine tris acid in DI H_2_O, (ii) C60-pyrrolidine tris acid on a mouse tissue section, (iii) an *E. coli* section without C60-pyrrolidine tris acid incubation, and (iv) a *B. subtilis* section without C60-pyrrolidine tris acid incubation. These data are shown in Supplementary Figure 2a–d. These results indicate positive cellular uptake of C60-derivatives in microorganisms, hence providing an opportunity to monitor the cellular activity *in situ* by further functionalization of C60.

**Figure 4:**
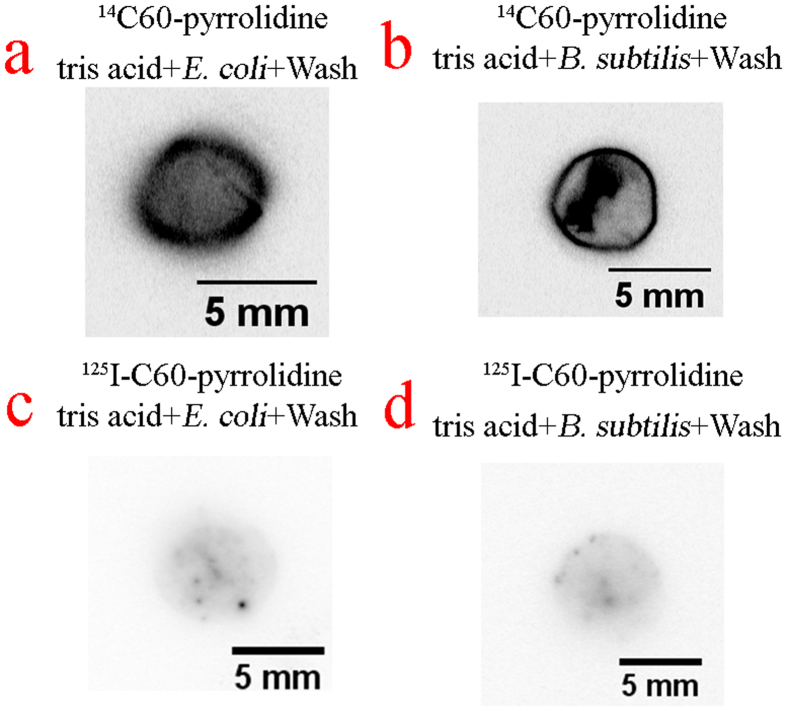
Cellular uptake of radiotracers labelled C60-pyrrolidine tris acid by microorganisms monitored by Autoradiography. (**a**) and (**b**) ^14^C60-pyrrolidine tris acid uptake by *E. coli (a)* and *B. subtilis(b)*. (**c**) and (**d**) ^125^I-C60-pyrrolidine tris acid uptake by *E. coli* (**d**) and *B. subtilis (d)*.

We then need to investigate whether C60-pyrrolidine tris acid can radiolabel microorganisms, which would thus provide a platform for imaging thick sections in an opaque environment. Even though radio tracers may have limitations in terms of resolution, having the probes for *in situ* imaging can motivate development of advanced instrumentation and design of novel experiments. C60-pyrrolidine tris acid is functionalized with ^14^C and ^125^I, and samples are incubated as before. Figure 4 shows β-radiation and γ-radiation emission from both *E. coli* (Fig. 4a,c) and *B. subtilis* (Fig. 4b,d), which are incubated with ^14^C- and ^125^I-labeled C60-pyrrolidine tris acid and imaged through autoradiography. The liquid scintillation data (Supplementary Figure 3) also shows a positive association of ^14^C-methylated C60-pyrrolidine tris acid by both *E. coli* and *B. subtilis* (about 12,000 counts per second). These results provide additional confirmation that C60-pyrrolidine tris acid can be internalized by microbes and visualized.

Finally, to investigate whether the number of washes or the incubation time has an impact on the C60 internalization, these parameters are changed, and the previous experiments are repeated. The rationale is that internalization might be a function of combined physical size, electrostatics, hydrophobicity, and diffusivity^45^,^46^,^47^. Results, shown in Supplementary Figures 4 and 5 and Supplementary Table 1 and 2, indicate that cellular (i) uptake is correlated with increased incubation time, and (ii) retention is not affected by the number of washes. These results have been obtained through unbiased and automated quantitative analysis, with an example shown in Supplementary Figure 6.

### Non-stickiness of C60-pyrrolidine tris acid to the substrates

Cell viability can be visualized by chemical staining of bacteria. For example, Life Technologies provides the LIVE/DEAD® BacLight™ Bacterial Viability Kit (L-7012), and Promokine provides an alternative assay known as PK-CA707-30002. These techniques rely on cell-penetrating dyes, such as SYTO9 or DMAO, to counterstain the nuclei in both live and dead cells, and non-cell-entering dyes, such as propidium iodide (PI) or EthD-III, to stain dead cells which have a damaged wall. However, these dyes and other antibodies for immunostaining are usually sticky to the natural environment (*e.g.*, soil, sand), creating significant background noise during visualization. Therefore, it is critical to evaluate the non-stickiness property of C60-derivatives. The degree of non-stickiness is evaluated using a variety of substrates (*e.g.*, glass beads, alumina, VWR sand, wild sand, and natural soil), with UV absorption and imaging that includes both fluorescence microscopy and use of an autoradiography/scintillation counter. These substrates cover a range of synthetic and natural environments for validation, while the readouts provide both bulk (*e.g.*, UV absorption) and spatial information (*e.g.*, imaging). The results are summarized below.

**Figure 5:**
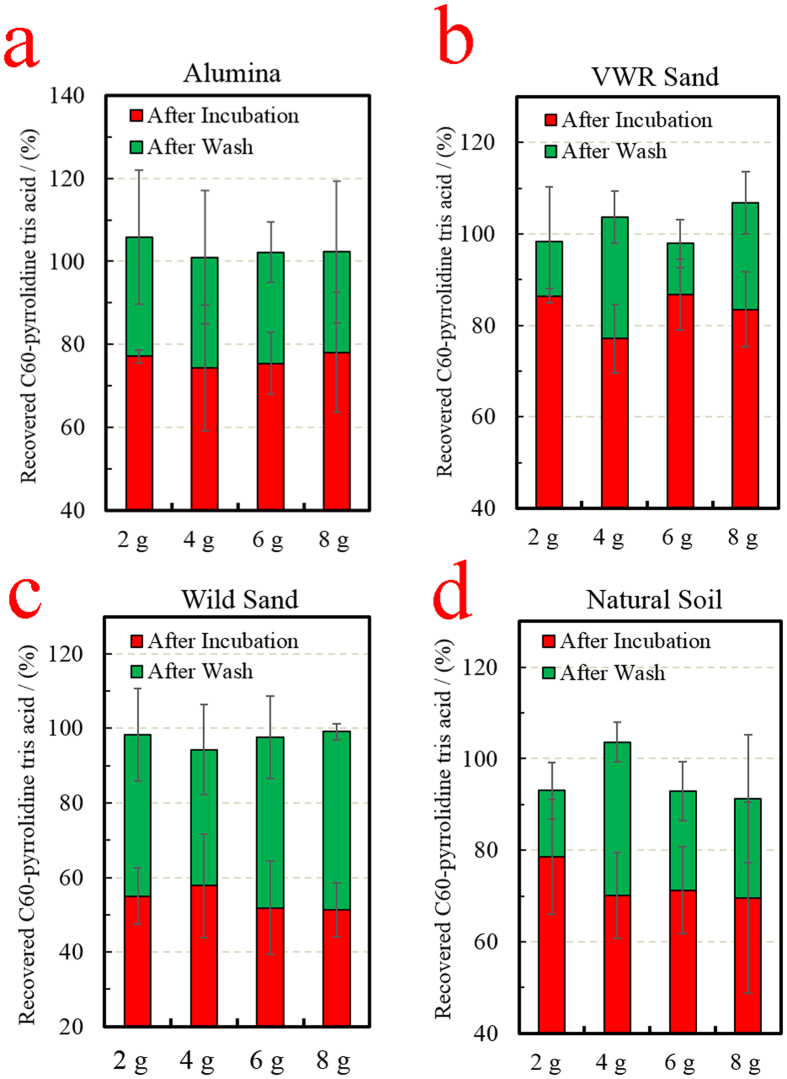
Non-stickiness of C60-pyrrolidine tris acid to multiple matrices, with increasing weights, monitored by Ultraviolet (UV) light absorption. The red regions indicate immediate recovery following incubation and the green regions indicate recovery following multiple washes. (**a-d**) Recovery of C60-pyrrolidine tris acid from alumina (**a**), VWR sand (**b**), wild sand (**c**), and natural soil (**d**). The results suggest that C60-pyrrolidine tris acid remains non-sticky to the various matrices.

The experimental setting for evaluating the non-stickiness property includes running H_2_O and a vacuum manifold to clear excess C60-pyrrolidine tris acid (Supplementary Figure 7). The stickiness of C60-pyrrolidine tris acid is quantified with multiple substrates, by measuring the UV absorption of filter-through at 335 nm, which is the specific absorption wavelength of C60^48^,^49^. With respect to synthetic substrates, alumina has been chosen as the representative substrate, since it is one of the top four components in the earth’s crust. Figure 5a indicates that more than 70% of the C60-pyrrolidine tris acid was recovered without H_2_O wash (red column) despite the mass of matrix. With respect to non-synthetic substrates, similar recovery rates are reported for homogenized VWR sand (>80%, Fig. 5b), Wild Sand (>60%, Fig. 5c), and Natural Soil (>70%, Fig. 5d), all without H_2_O wash (red column). The remainder of C60-pyrrolidine tris acid is fully recovered from the matrices, following H_2_O rinses (green columns in Fig. 5a–d). These results indicate that C60-pyrrolidine tris acid is not sticky to the natural environment. The rationale for requiring a second wash is due to the meso-porous architecture of the matrices that trap C60-derivatives.

**Figure 6:**
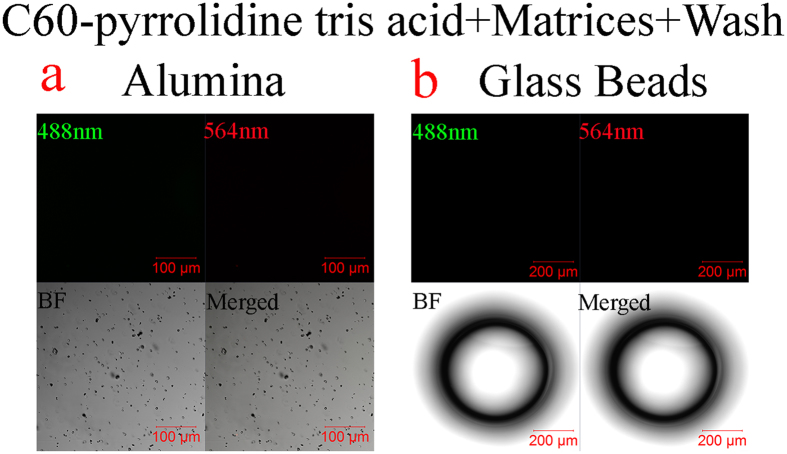
Non-stickiness of C60-pyrrolidine tris acid monitored by Confocal Laser Scanning Microscopy following several washes. Fluorescence microscopy indicates that fBSA labelled C60-pyrrolidine tris acid has no adherence to alumina (**a**) and glass beads (**b**), which are clearly present in bright field microscopy.

**Figure 7:**
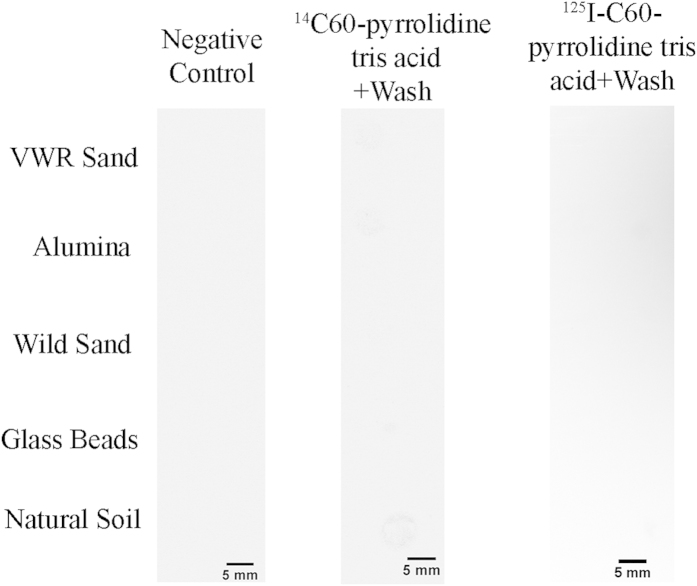
Non-stickiness of radiotracers ^14^C- and ^125^I-labeled C60-pyrrolidine tris acid, on several matrices, monitored by Autoradiography following several washes. The left column shows the background radiation. The middle and right columns indicate that neither β-radiation nor ϒ-radiation are detected by autoradiography after several washes.

To investigate the non-stickiness property of C60-pyrrolidine tris acid spatially, several experiments have been designed. (i) fBSA-labeled C60-pyrrolidine tris acid is evaluated against non-auto-fluorescent matrices such as glass beads and alumina (Fig. 6). Using fluorescence microscopy, each substrate emits an initial fluorescent signal after incubation with C60-pyrrolidine tris-fBSA, with the signal being lost following multiple H_2_O washes, thus providing confirmation that C60-pyrrolidine was not sticky to the natural environment. (ii) fBSA-labeled C60-pyrrolidine tris acid is evaluated against matrices such as VWR sand, wild sand, and natural soil (Supplementary Figure 8). However, these matrices are auto-fluorescent and mask fluorescent probes, making it difficult to visualize the fluorescent-labeled C60. (iii) Further validation by autoradiography and liquid scintillation is pursued in all substrates (*e.g.*, pretreated VWR sand, glass beads, alumina, wild sand, and natural soil). Figure 7 indicates that neither β- nor γ-radiation are detected from incubated matrices (middle and right columns) after 6 H_2_O rinses. Therefore, we conclude that C60-derivatives are non-sticky to the natural environment and can be removed entirely by H_2_O washes.

### Uptake of functionalized C60-pyrrolidine tris acid in microbes embedded in soil matrices

**Figure 8:**
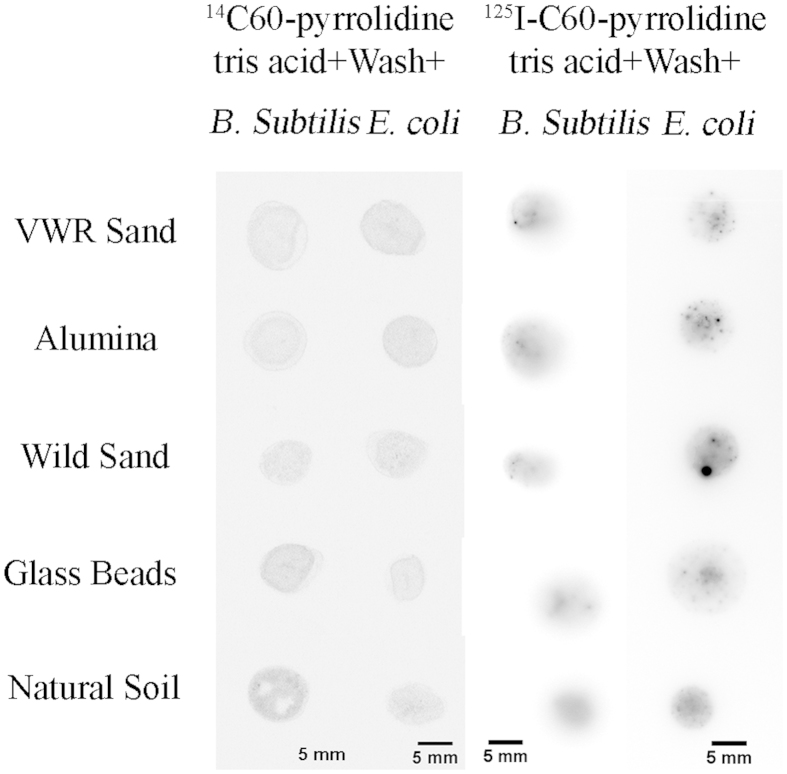
Cellular uptake of microorganisms embedded in different matrices and monitored by Autoradiography following several washes. Left and right columns indicate uptake by ^14^C- and ^125^I- labelled C60-pyrrolidine tris acid on *B. subtilis* and *E. coli*, respectively. The data suggests a residual signal that is presumably due to the uptake of microorganisms, since Fig. 7 indicates non-stickiness to the same substrates.

**Figure 9:**
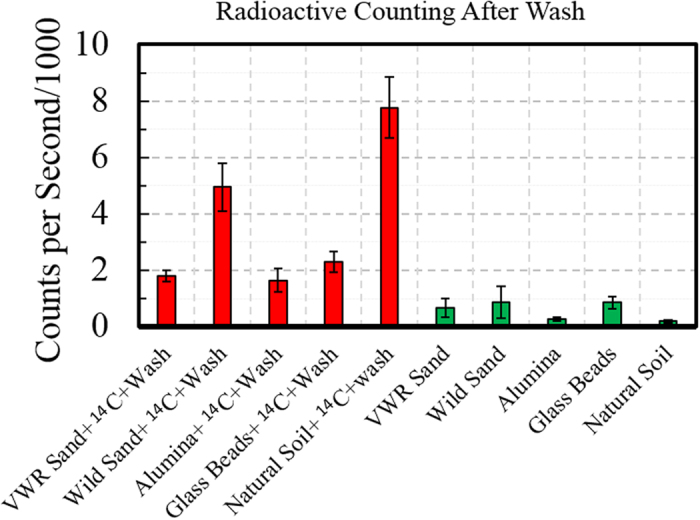
Non-stickiness of ^14^C-labelled C60-pyrrolidine tris acid with five different matrices, monitored by Liquid Scintillation Count (LSC) following several washes. Red columns show similar level of LSC from incubation and washout of VWR sand, wild sand, alumina, glass beads, and natural soil with ^14^C labelled- C60-pyrrolidine tris acid. Green bars show the background LSC on the same substrates.

Natural soil is a complicated biomaterial, hosting thousands of microorganisms with intrinsic organic and inorganic matters that hinder probe delivery. ^14^C- and ^125^I-radiolabeled C60-pyrrolidine tris acid are incubated with a mixture of soil and microorganisms and then washed to remove excess probes as before. The autoradiography, shown in Fig. 8, indicates strong β- and γ− radiation from the mixture of soil and microorganisms. Comparison of this result with both (i) Fig. 4a,b, which indicated association with microorganisms, and (ii) Fig. 7a,b, which indicated non-stickiness to the matrix, suggests that radiotracers can label microbes in their native environment. Moreover, interesting observations are made when β-radiation is quantified using a liquid scintillation counter, comparing both control and treated matrices with ^14^C-labeled C60-pyrrolidine tris acid. All control matrices (*e.g.*, background) show around 1,000 counts per second (Fig. 9, green columns), while ^14^C-labeled C60-pyrrolidine tris acid incubated with glass beads, alumina, VWR sand, wild sand, and natural soil show 2,000, 2,000, 2,000, 4,000, and 6,000 counts per second respectively (Fig. 9, red columns). These results suggest that the natural microorganisms in wild sand and natural soil have successfully taken up ^14^C-labeled C60-pyrrolidine tris acid, which accounts for the increased number of counts per second.

### Differentiation of Live and Dead Microorganisms

To investigate differentiation between live and dead cells, C60 is functionalized with four different amino acids and then screened. This approach is motivated by the fact that different microorganisms have varying preferences for a specific amino acid^50^. C60-pyrrolidine tris acid is functionalized with the amino acids glycine, tryptophan, arginine, and cysteine. In particular, cysteine functionalized C60-pyrrolidine tris acid allows for the differentiation of live and dead cells in the presence of the substrate matrix.

**Figure 10:**
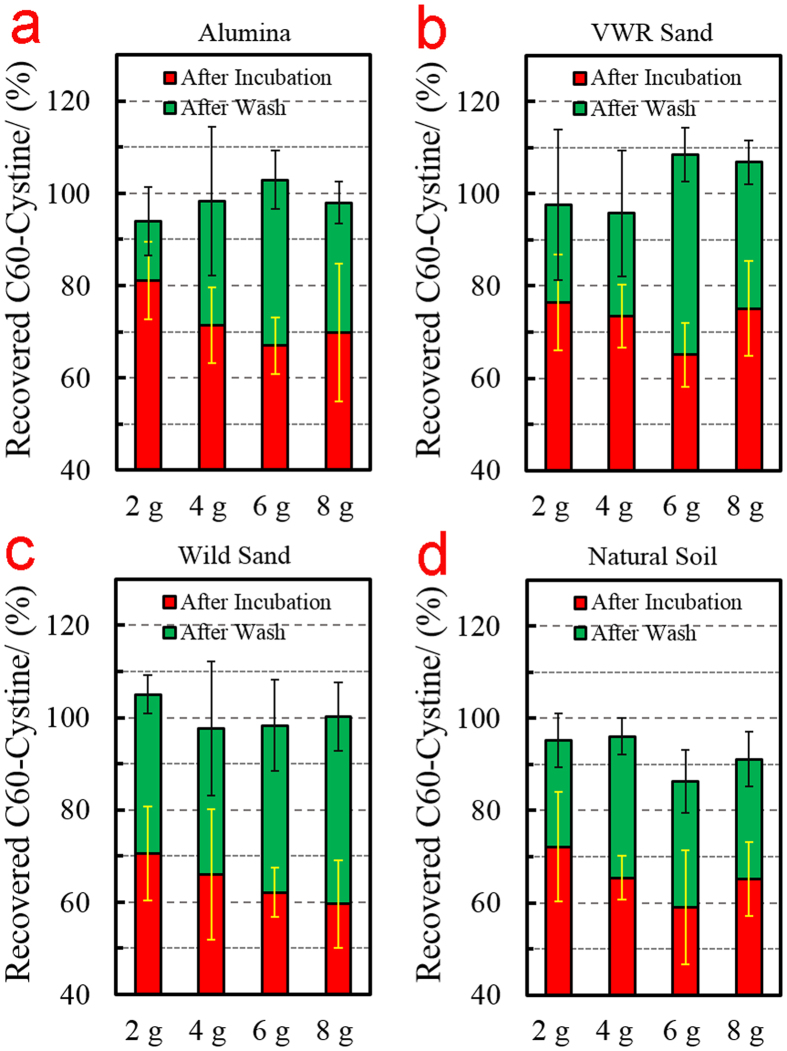
Non-stickiness of C60-pyrrolidine tris-Cysteine to multiple soil matrices monitored by Ultraviolet light absorption. Recovery of C60-Cysteine from alumina (**a**), VWR sand (**b**), Wild Sand (**c**) and Natural Soil (**d**) of different mass indicate that the newly synthesized C60-pyrrolidine tris-Cysteine remains non-sticky to multiple matrices. The red regions indicate immediate recovery following incubation, and the green regions indicate recovery following multiple washes.

To validate non-stickiness to the matrix, both UV absorption and microscopy are utilized. Figure 10 shows a trend for recovering C60-pyrrolidine tris-cysteine from alumina (Fig. 10a), VWR sand (Fig. 10b), wild sand (Fig. 10c), and natural soil (Fig. 10d), all of which show results comparable to those previously reported (Fig. 5). More than 60% of C60-pyrrolidine tris-cysteine is removed from alumina, wild sand, and natural soil, without wash (red column). The rest of C60-pyrrolidine tris-cysteine is fully recovered with one additional H_2_O wash (green column).

**Figure 11:**
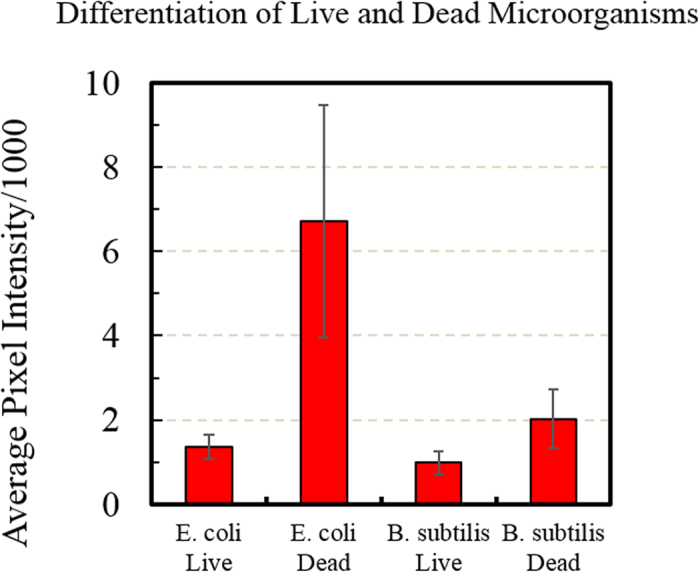
Cellular uptake C60-Cystine for differentiating live and dead microorganisms (*E. coli* and *B. subtilis*), quantified by analyzing images from confocal microscopy. Live microorganisms show a significantly lower uptake than the dead microorganisms. The uptake is quantified by the average pixel intensities of micoorganisms over the background.

To differentiate and quantify live and dead microorganisms, fluorescence microscopy is used. The results indicate a significantly lower signal for live cells than for dead cells for both *E. coli* and *B. subtilis*, as shown in Fig. 11. Both live *E. coli* and *B. subtilis* show a base line fluorescence signal of approximately 1,000 (in pixel intensity), whereas dead *E. coli* and *B. subtilis* show a significantly higher signals, of approximately 6,500 and 2,000, respectively. This observation is potentially due to the fact the dead cells have a leaky structure, which allows more C60-pyrrolidine tris-cysteine to cross their cellular membrane. In addition, the cell wall of dead *B. subtilis* (Gram-positive, and with a thicker cell wall) may not be as leaky as dead *E. coli*; thus, dead *E. coli* shows a higher fluorescence signal than *B. subtilis*. The net result is that functionalized C60-pyrrolidine tris acid can differentiate cellular states.

## Conclusions

This paper demonstrates that C60-derivatives have the potential to visualize microorganisms in their natural environments. Through a series of well-designed experiments, it is shown that the C60-derivatives (i) are capable of internalization within Gram-positive and Gram-negative microorganisms in their natural environments; (ii) maintain non-stickiness to the soil matrix; and (iii) are able to differentiate between live and dead microbial cells. These findings, coupled with C60’s internalization mechanism, provide the rationale for further development of multiplatform C60 (*e.g.*, with fluorescent and radiotracer reporters) for monitoring the bioactivity of microbial communities. Such a system can also be used to monitor and track infiltration of pathogens in their host environment, *i.e.*, pre-labeling pathogens, injecting them into their host, and monitoring microbial life cycle using functional imaging. The technology will empower a wide array of applications (*e.g.*, portable sensor chips) for studying microbial systems in their native environment, with high sensitivity and a low C60 concentration of 5–10 μg/mL.

## Materials and Methods

### Conjugation of Fluorescent Bovine Serum Albumin/Amino Acids

C60-pyrrolidine tris acid (1 mg) (Sigma) was dispersed in 0.5 mL of 2-(N-morpholino)ethanosulfonic acid (pH 5.6) (MES) (Sigma) buffer under sonication for 30 min at ambient conditions. 0.25 mL of 1-(3-dimethylaminopropyl)-3-ethylcarbodiimide hydrochloride (0.4 mol/L) (EDC) (Sigma) and 0.25 ml of N-hydroxysuccinimide (0.2 mol/L) (NHS) (Sigma) in MES solution were added to the activated carboxylate groups^37^,^51^,^52^. The mixture was washed with PBS and centrifuged at 12,000 *g* for 30 min in a 5 kDa molecular weight cutoff centrifugal filter (Millipore) for 5 times, to remove EDC and NHS. 2 mg of fluorescent bovine serum albumin (fBSA) (Sigma)/cysteine (Acros) were then added into the C60-pyrrolidine tris acid/PBS solution at 4 ^o^C overnight for conjugation. The mixture was finally washed with PBS and centrifuged in a micro-centrifuge tube to remove un-conjugated protein/cysteine in supernatant; this centrifugation was repeated 5 times at 12,000 rcf for 30 min^37^. The C60-pyrrolidine tris acid-fBSA/cysteine pellet was collected and stored at −20 ^o^C until use.

For further labeling C60-pyrrolidine tris-cysteine with Atto565 NHS Easter (Sigma), C60-pyrrolidine tris-cysteine (1 mg) were suspended in 1 mL EDC/MES solution (0.1 mol/L); Atto 565 NHS Easter (20 μL) was then added at 4 ^o^C overnight for conjugation. The mixture was finally washed with PBS and centrifuged in a micro-centrifuge tube to remove un-conjugated Atto565 in supernatant; the centrifugation was repeated 5 times at 12,000 rcf for 30 min^37^. The C60-pyrrolidine tris acid-cysteine-Atto565 pellet was collected and stored at −20 ^o^C until use.

### Radio-labeling of ^14^C and ^125^I to C60-pyrrolidine tris acid

^14^C labeling was carried out on dry ice and under argon in the fume hood. C60-pyrrolidine tris acid (100 μg) was dispersed in 1 mL dimethylformamide (DMF) (Sigma) homogeneously in a 15 mL conical tube. Potassium carbonate (1 mg) was added as base. Then, 50 μL ^14^C-methyl iodide (1.85 MBq) was added, and the tube was capped tightly. Until this point, H_2_O must be avoided. The tube was transferred at room temperature to allow the mixture to react for 1 hour with periodic agitation (every 10 min)^53^. Potassium carbonate was then removed while excess DMF and unreacted ^14^C-methyl iodide were evaporated using an oil bath. Next, ^14^C labeled C60-pyrrolidine tris acid was suspended in 1mL DMF, and its radioactivity was counted in the liquid scintillation counter. The radiochemical yield was calculated as ~20% as following:


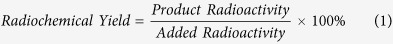


^125^I-labeling was performed under ambient conditions in a 1.5 mL microcentrifuge tube. C60-pyrrolidine tris acid (100 μg) was dispersed in 1 mL phosphate buffer (pH 8.0). 5 μL of Na^125^I (0.56 MBq) and 5 μL of N-chlorosuccinimide (10 mg/mL in DI H_2_O) were then added. The reaction was allowed to continue for 20 min with periodic shaking (every 5 min). The reaction was then quenched by adding 10 μL of sodium bisulphite (10 mg/mL in DI H_2_O) (Sigma) and 10 μL of NaI (10 mg/mL in DI H_2_O) (Sigma). The mixture was centrifuged at 20,000 rcf for 15 min to pellet the ^125^I-labeled C60-pyrrolidine tris acid. The pellet was washed with DI H_2_O and re-pelleted 5 times at 20,000 rcf for 15 min until the radioactivity of the wash reached normal level. The yield was calculated to be ~20% according by Equation 1.

### Fluorescence Microscopy

A Zeiss 710 confocal microscopy systemfluorescence microscope was utilized to (i) profile microbial and matrix autofluorescence, and microbial uptake, (ii) differentiate live and dead cells, and (iii) evaluate adherence to the matrix. Depending upon the use-case, samples were imaged with either a Zeiss Plan-Neofluar 10x/0.30 objective or a Plan-Apochromat 63x/1.40 Oil immersion objective. The soil matrices were imaged with the 10x objective, while microbial species were imaged and subsequently quantified with the 63x objective. The excitation filters were set at 488 and 561 nm, and the emission filters were set to receive signals between 493–556 nm and 597–700 nm, respectively. The laser intensity was set at 20% to excite fBSA and matrix autofluorescence. A twin-gate main beam splitter with two wheels and each wheel having 10 filter positions (*e.g.,* 100 possible combinations) was used to separate excitation and emission beams. The pinhole was set at 600 μm to receive as many photons as possible.

To examine the auto-fluorescence of microorganisms, 1.0 mL of bacterial cells (OD_630 _= 0.6) were pelleted by centrifugation at 5,000 rcf to remove the old medium, then suspended in 1.0 mL fresh medium. 5 μL of the suspension was then mounted onto a glass slide under a coverslip, sealed with nail polish hardener. To examine the retention of C60-pyrrolidine tris acid within the microorganisms, 1.0 mL of bacterial cells (OD_630 _= 0.6) were pelleted at 5,000 rcf to remove the old medium. Cells were suspended in fresh medium in a 1.5 mL micro-centrifuge tube and incubated in the dark at with C60-pyrrolidine tris acid-fBSA (5 μg/mL) predetermined times: 5 min, 30 min, 1 hour, and 2 hours. The tube was then centrifuged at 5,000 rcf to remove the non-penetrated C60-pyrrolidine tris acid-fBSA, and the microorganisms were suspended in fresh medium. 5 μL of the suspension was then mounted onto a glass slide under a coverslip, sealed with nail polish hardener.

To examine the retention of C60-pyrrolidine tris acid within the microorganisms after wash, the pellets were washed with DI H_2_O, and the C60-pyrrolidine tris acid-fBSA internalized microorganisms were re-pelleted at 5,000 rcf for 5 min in the dark. This was repeated 6 times. 5 μL of the suspension was then mounted onto a glass slide under a coverslip, sealed with nail polish hardener. The fluorescent images were taken using a Zeiss 710 confocal microscopy system.

To differentiate live and dead microorganisms, freshly prepared C60-pyrrolidine tris-cysteine-Atto565 conjugate was used. First, live or dead microorganisms (*E. coli* and *B. subtilis*) (dead cells were obtained by incubating live cells at 90 ^o^C for 30 minutes) were incubated in the dark with C60-cysteine-Atto565 (10 μg/mL) for 30 min. Second, both live and dead microorganisms were pelleted at 5,000 rcf for 5 min, and the supernatant was carefully removed. Third, the live and dead microorganism pellets were washed with DI H_2_O, then re-pelleted. Finally, 5 μL of the final suspensions for live and dead microorganism were mounted onto two respective glass slides under coverslips, sealed with nail polish hardener. The prepared slides were imaged using a Zeiss 710 confocal microscope under identical microscopic conditions (*e.g.*, laser intensity, pinhole setting, gain). The images obtained with the Zeiss 710 confocal microscope were not modified by other software and were analyzed by ImageJ software. The process of the image analysis is: (I) The fluorescent signal of the microorganisms (single cell or cluster of cells) were selected by the freehand selection tool and the background signal was subtracted. (II) The mean intensity of the selected area was measured by ImageJ. (III) The average signal was then calculated by averaging the mean intensity of multiple selected areas.

To examine the adherence of C60-pyrrolidine tris acid to the matrix, several substrates were used. These are VWR Sand (VWR), alumina (Acros), glass beads, wild sand (collected at the Aquatic Park Innovation Center, Berkeley CA), and natural soil (collected at the backyard garden of Aquatic Park Innovation Center, Berkeley CA, courtesy of the building management). These substrates (0.2 g) were incubated in the dark with 1 mL C60-pyrrolidine tris acid-fBSA (5 μg/mL) for 30 min. Each substrate was then washed 6 times with 1 mL DI H_2_O (10 min incubation). Next, 10 grains of substrate particle were carefully dried of liquid, and then mounted onto glass slides with nail polish hardener as a sealant. The fluorescent images were taken by Zeiss 710 confocal microscope.

### Retention of C60-derivatives from multiple substrates

To examine the retention of C60-derivatives in multiple substrates with different masses (2 g, 4 g, 6 g, and 8 g), we incubated each substrate (*e.g.*, wild sand, natural soil, VWR sand) with C60-pyrrolidine tris acid at a concentration of 5 μg/mL on a layer of filter paper (with an average pore size of 25 μm). After 30 min of incubation, vacuum suction was applied, and the first filter-through of C60-pyrrolidine tris acid was collected. The residue on the filter paper was washed with DI H_2_O (10 min of incubation), and the filter-through of C60-pyrrolidine tris acid was collected for the second time. Both filter-throughs were measured by UV-vis spectrometer at 335 nm^48^,^49^. The recovery of C60-pyrrolidine tris acid was determined as following:


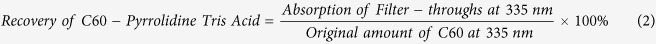


### Transmission Electron Microscopy (TEM)

TEM was used to examine whether the retention of C60-pyrrolidine tris acid was localized within the cytosol. Microorganisms were incubated with C60-pyrrolidine tris acid (5 μg/mL) for 30 min. Cells were pelleted by centrifugation at 5,000 rcf (5 min) and washed 5 times with DI H_2_O. Microorganisms were fixed in 2.5% glutaraldehyde/PBS solution for 30 min. The cells were then pelleted by centrifugation at 5,000 rcf for 5 min. The cell pellet was placed in fresh 2.5% glutaraldehyde/PBS solution at 4 ^o^C overnight. Next, cell pellets were dehydrated by a series of acetone treatments (30% for 15 min, 50% for 15 min, 70% for 15 min, 90% for 15 min and a final treatment at 100% for 30 min, and repeated 3 times), embedded in resin (2:1 mix of propylene oxide:resin for 1 hour, 1:1 mix of propylene oxide:resin for 1 hour, 1:2 mix of propylene oxide: resin for 1 hour, 100% resin overnight, and change to fresh resin for 1 h), incubated for 24 h at 37 ^o^C, sectioned (60 nm in thickness), and imaged using a Tecnai 12 TEM.

### Liquid Scintillation Counter

To examine the retention of C60-pyrrolidine tris acid within multiple substrates, we incubated each substrate (0.2 g) in the dark with 1 mL of ^14^C labeled C60-pyrrolidine tris acid (5 μg/mL) for 30 min. We pelleted the substrate without microorganisms at 5,000 rcf and washed those pellets 6 times with 1 mL DI H_2_O (10 min incubation). The final pellets were added to 3 mL of scintillation cocktail. The data were recorded using a liquid scintillation counter (Perkin Elmer).

### Autoradiography

To examine the retention of C60-pyrrolidine tris acid in *B. subtilis* and *E. coli* with multiple substrates, we incubated each substrate (0.2 g) in the dark with 1 mL ^14^C- and ^125^I-labeled C60-pyrrolidine tris acid (5 μg/mL) for 30 min. We pelleted substrates both with and without microorganisms at 5,000 rcf and washed those pellets 5 times with 1 mL fresh DI H_2_O (10 min incubation). The final pellet was dispersed in 0.1 mL fresh LB broth. 1/10 of the volume was carefully transferred onto a piece of plastic wrap, under which was a phosphor imager film. After 24 hours of sitting in complete darkness, the film was transferred into a Cyclone Plus Phosphor Imager (Perkin Elmer) for imaging.

### Cell culture

*E. coli* (Invitrogen) and *B. subtilis* (ATCC) were cultured in LB Broth at 37 ^o^C with constant shaking. *E. coli* and *B. subtilis* were not used until OD_630_ reached 0.6.

### Ethics statement

The natural soil (100 g in total) collected from the backyard of the Aquatic Innovation Center was a courtesy from the building management, Wareham Development. There is no special regulation in collecting a small amount of beach sand (100 g in total) from Aquatic Park, Berkeley CA, where a recreational area involves a wide range of human activities, etc. boating, biking, and hiking. Our research does not include any field study or endangered species.

### Statistical analysis

Four samples were analyzed at each condition. The data in the graphs are represented by their mean ± standard deviation (SD).

## Additional Information

**How to cite this article**: Cheng, Q. *et al.* Functionalized Buckyballs for Visualizing Microbial Species in Different States and Environments. *Sci. Rep.* **5**, 13685; doi: 10.1038/srep13685 (2015).

## Supplementary Material

Supplementary Information
