# Supplementary material for: Functionalized Buckyballs for Visualizing Microbial Species in Different States and Environments
Source: Sci Rep. 2015 Sep 8;5:13685. doi: 10.1038/srep13685 (PMC4561912; doi:10.1038/srep13685)
Supplement: Supplementary Information [file srep13685-s1.pdf]

Supporting Information

Functionalized Buckyballs for Visualizing Microbial Species in Different States and Environment

Qingsu Cheng[1,2], *Ashwin Aravind*[1], *Mathew Buckley*[1], *Andrew Gifford*[3], *and Bahram Parvin*[1,2]*

1 Department of Electrical and Biomedical Engineering, University of Nevada, Reno, 1664 N Virginia Street, Reno NV, 89503, USA

2 Life Science Division, Lawrence Berkeley National Laboratory, 1 Cyclotron Road, MS-977, Berkeley CA, 94720, USA.

3 Brookhaven National Laboratory, Bioscience Department, Bldg490, Upton, NY11973, USA.

Corresponding author:
Bahram Parvin
E-mail: b_parvin@lbl.gov

Supplementary Table: Quantified signal intensity for Supplementary Figure 4

	Microorganism Signal Intensity	Background Signal Intensity
Supplementary Figure 4a	273±71	40±8
Supplementary Figure 4b	345±56	120±10
Supplementary Figure 4c	372±103	70±15
Supplementary Figure 4d	529±105	150±13
Supplementary Figure 4e	654±54	224±48
Supplementary Figure 4f	860±123	257±32
Supplementary Figure 4g	1128±210	321±22
Supplementary Figure 4h	1323±239	284±41

Supplementary Table 2: signal intensity for Supplementary Figure 5

	Microorganism Signal Intensity	Background Signal Intensity
Supplementary Figure 5a	579±167	40±15
Supplementary Figure 5b	942±167	225±34

Figure Captions
Supplementary Figure 1. Autofluorescence of *B. subtilis* and *E. coli* monitored with a confocal microscope indicate no signals. (a) and (b) Autofluorescence background for *B. subtilis* (a) and *E. coli (b)* with 488nm excitation. (c) and (d) Bright field images of *B. subtilis* (c) and *E. coli* (d) indicate presence of microorganisms. (e) and (f) Merged bright field and autofluorescence provides an additional evidence for absence of any signal.
Supplementary Figure 2. Positive and negative controls for C60-pyrrolidine tris acid monitored with transmission electron microcopy. C60-pyrrolidine tris acid is clearly present in (a) DI H_2O and (b) a tissue section from mouse as background. The background, without C60-pyrrolidine tris acid, is void of any signal in (a) *E. Coli* and (b) *B. subtilis*.
Supplementary Figure 3. Uptake of ^{14}C-labelled C60-pyrrolidine tris acid monitored by Liquid Scintillation Count for (a) *E. coli* and (b) *B. subtilis*.
Supplementary Figure 4. Time course experiments for uptake of C60-pyrrolidine tris acid-fBSA, monitored by confocal microscopy, for *E. coli (*a, c, e, and f) or *B. Subtilis* (b, d, f, and h) indicate time-dependency.
Supplementary Figure 5. Retention of C60-pyrrolidine tris acid-fBSA, monitored by confocal microscopy, after 6 rinses for (a) *E. coli* and (b) *B. Subtilis.*
Supplementary Figure 6. Steps in quantification of fluorescent images captured through confocal microscopy for supplementary tables 1-2: (a) Enhanced image for visualization; (b) segmented microbes followed by connected components; and (c) extracted boundaries for each segmented microbe.
Supplementary Figure 7. Experimental setup for measuring UV absorption: (a) Incubate C60-pyrrolidine tris acid with the substrate for 30 mins, (b) apply a vacuum manifold to C60-pyrrolidine tris acid and collect filtrate, (c) add fresh water to the substrate again and incubate for another 30 mins, and (d) apply a vacuum manifold to wash water and collect filtrate again.
Supplementary Figure 8. Auto-fluorescence of (a) VWR sand, (b) wild sand, and (c) natural soil, all monitored by confocal microscopy with 488nm excitation.

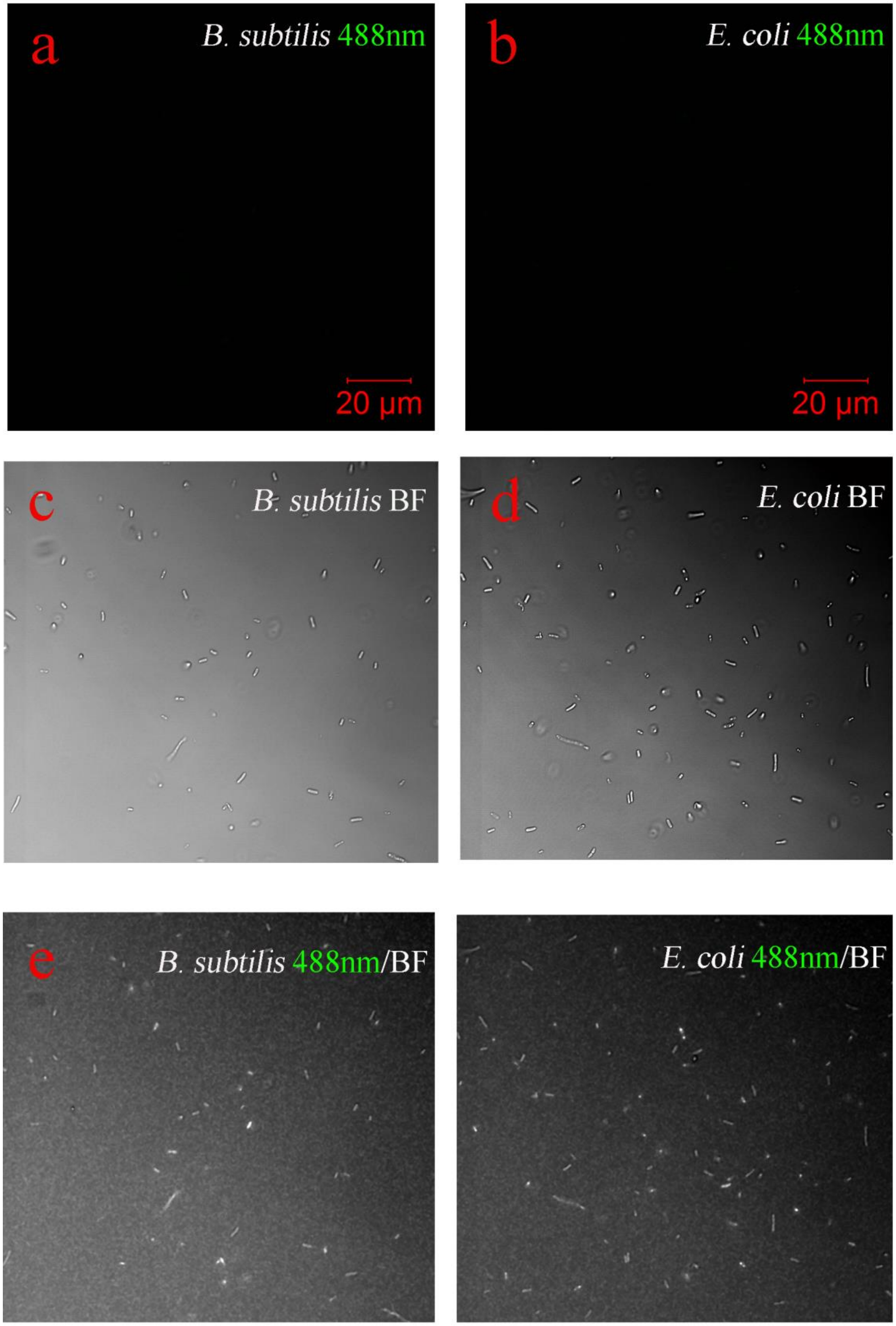
a
B. subtilis 488nm
20 μm
b
E. coli 488nm
20 μm
c
B. subtilis BF
d
E. coli BF
e
B. subtilis 488nm/BF
E. coli 488nm/BF

Supplementary Figure 1. Autofluorescence of *B. subtilis* and *E. coli* monitored with a confocal microscope indicate no signals. (a) and (b) Autofluorescence background for *B. subtilis* (a) and *E. coli (b)* with 488nm excitation. (c) and (d) Bright field images of *B. subtilis* (c) and *E. coli* (d) indicate presence of microorganisms. (e) and (f) Merged bright field and autofluorescence provides an additional evidence for absence of any signal.

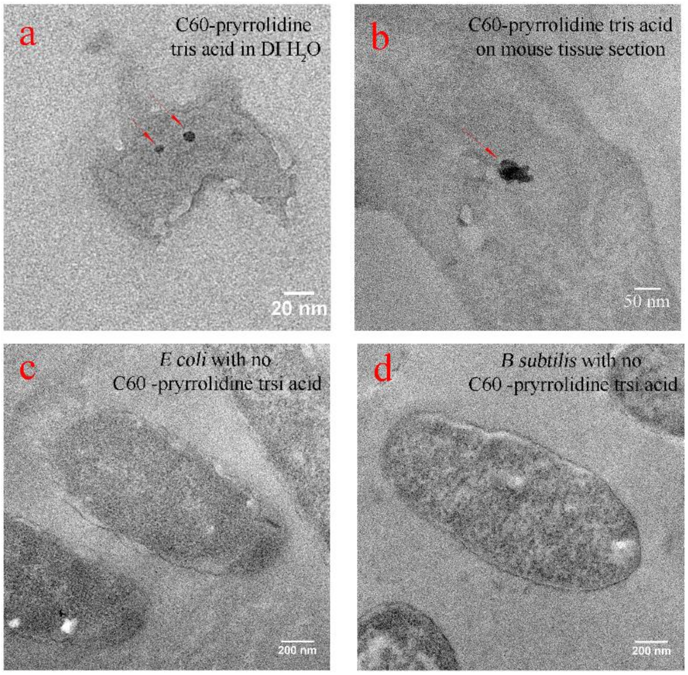

Supplementary Figure 2. Positive and negative controls for C60-pyrrolidine tris acid monitored with transmission electron microcopy. C60-pyrrolidine tris acid is clearly present in (a) DI H_2O and (b) a tissue section from mouse as background. The background, without C60-pyrrolidine tris acid, is void of any signal in (a) *E. Coli* and (b) *B. subtilis*.

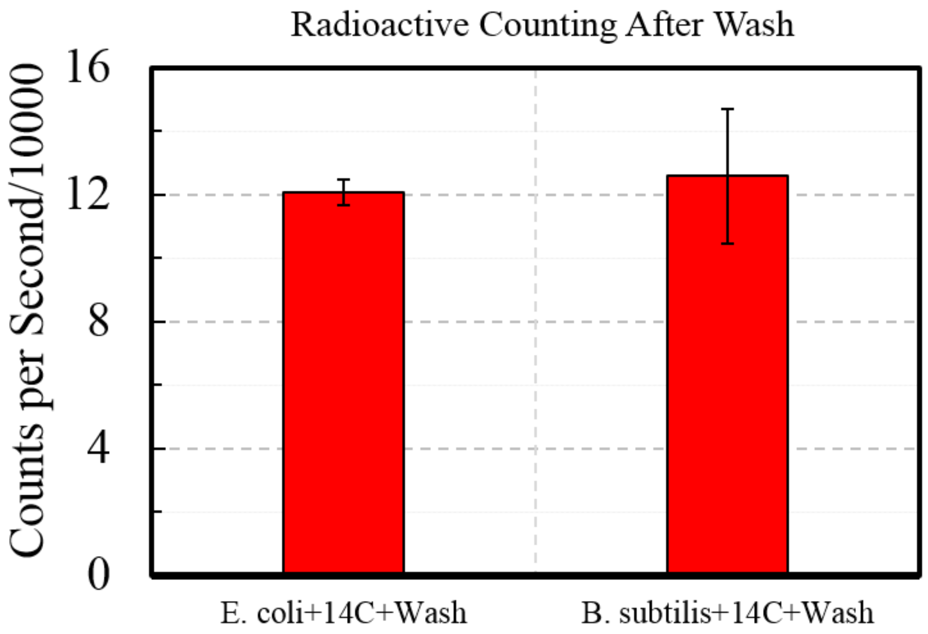

Supplementary Figure 3. Uptake of ^{14}C-labelled C60-pyrrolidine tris acid monitored by Liquid Scintillation Count for (a) *E. coli* and (b) *B. subtilis*.

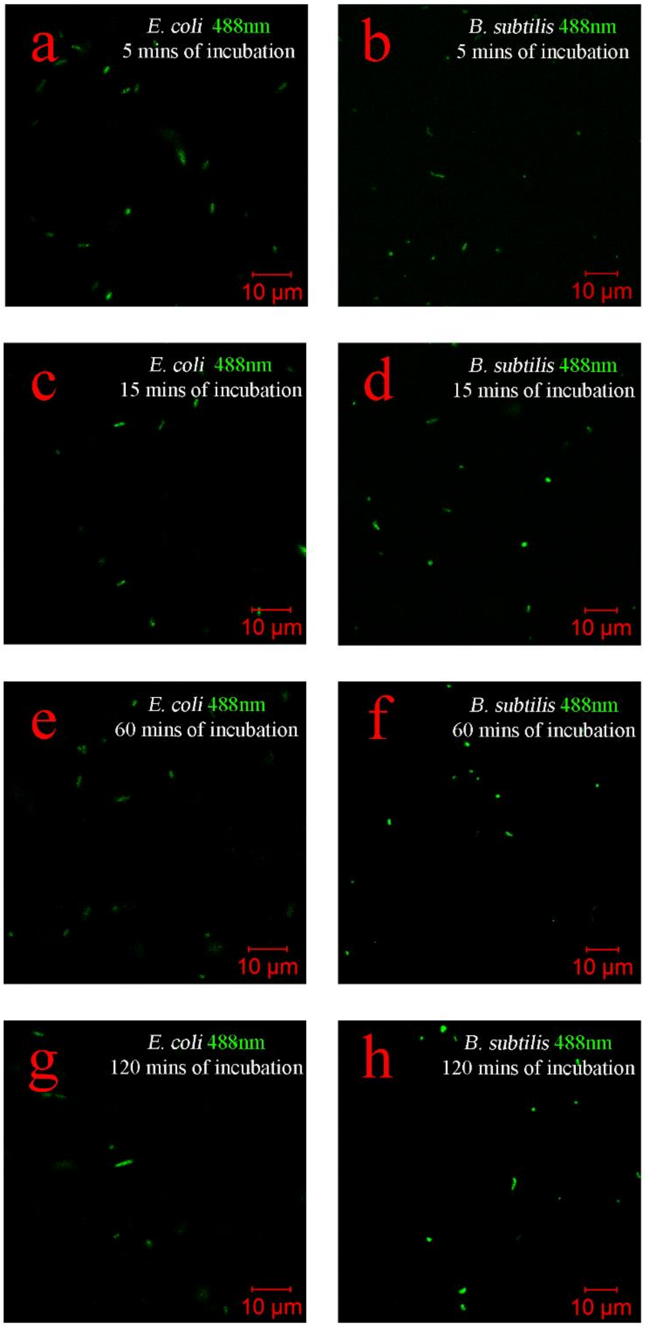

Supplementary Figure 4. Time course experiments for uptake of C60-pyrrolidine tris acid-fBSA, monitored by confocal microscopy, for *E. coli (*a, c, e, and f) or *B. Subtilis* (b, d, f, and h) indicate time-dependency.

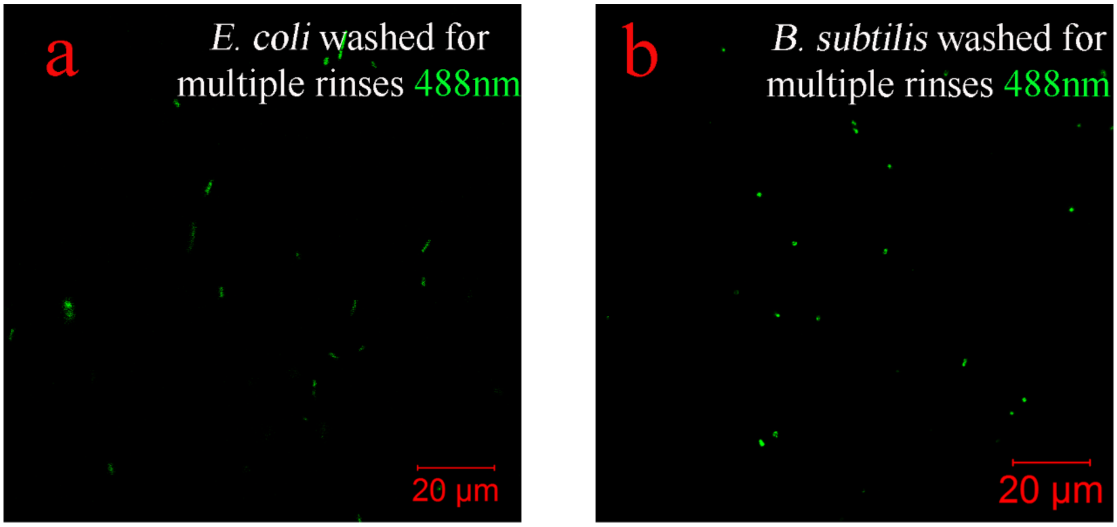

Supplementary Figure 5. Retention of C60-pyrrolidine tris acid-fBSA, monitored by confocal microscopy, after 6 rinses for (a) *E. coli* and (b) *B. Subtilis.*

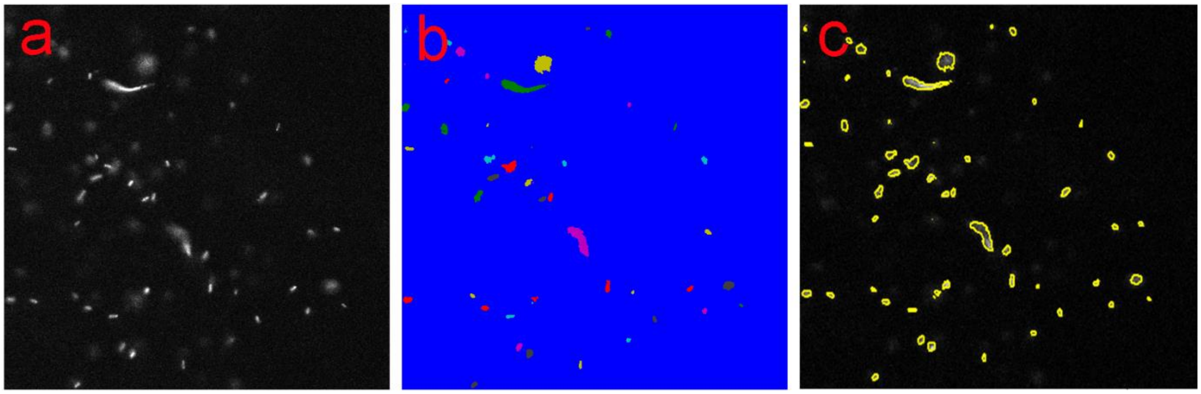

Supplementary Figure 6. Steps in quantification of fluorescent images captured through confocal microscopy for supplementary tables 1-2: (a) Enhanced image for visualization; (b) segmented microbes followed by connected components; and (c) extracted boundaries for each segmented microbe.

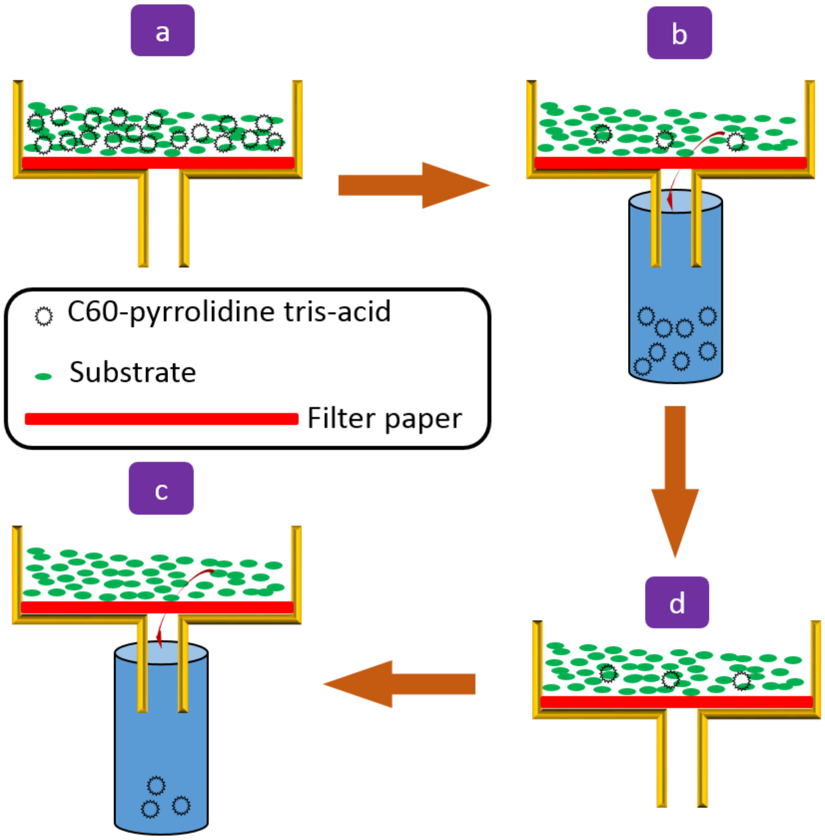

Supplementary Figure 7. Experimental setup for measuring UV absorption: (a) Incubate C60-pyrrolidine tris acid with the substrate for 30 mins, (b) apply a vacuum manifold to C60-pyrrolidine tris acid and collect filtrate, (c) add fresh water to the substrate again and incubate for another 30 mins, and (d) apply a vacuum manifold to wash water and collect filtrate again.

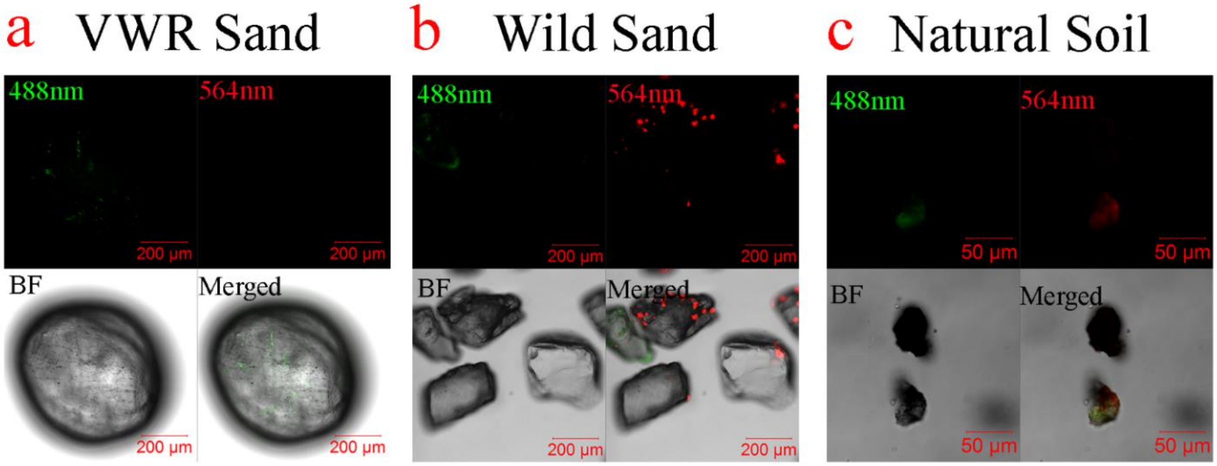

Supplementary Figure 8. Auto-fluorescence of (a) VWR sand, (b) wild sand and (c) natural soil, all monitored by confocal microscopy with 488nm excitation.
